# Complete Genome Sequences of 11 *Bordetella pertussis* Strains Representing the Pandemic *ptxP3* Lineage

**DOI:** 10.1128/genomeA.01394-15

**Published:** 2015-11-25

**Authors:** Marieke J. Bart, Han G. J. van der Heide, Anne Zeddeman, Kees Heuvelman, Marjolein van Gent, Frits R. Mooi

**Affiliations:** a Department of Pediatrics, Laboratory of Pediatric Infectious Diseases, Radboud University Medical Center, Nijmegen, The Netherlands; b Centre for Infectious Diseases Control (CIb), National Institute for Public Health and the Environment (RIVM), Bilthoven, The Netherlands

## Abstract

Pathogen adaptation has contributed to the resurgence of pertussis. To facilitate our understanding of this adaptation we report here 11 completely closed and annotated *Bordetella pertussis* genomes representing the pandemic *ptxP3* lineage. Our analyses included six strains which do not produce the vaccine components pertactin and/or filamentous hemagglutinin.

## GENOME ANNOUNCEMENT

*Bordetella pertussis* is the causative agent of pertussis or whooping cough, a respiratory disease which is most severe in young unvaccinated infants. After the introduction of vaccination in the 1950s, there was a steep decline in disease incidence. However, in the 1990s pertussis resurged. The increase in notifications was initially attributed to increased awareness and improved diagnostics. However, later it became clear that the pertussis resurgence was mainly due to suboptimal vaccines and pathogen adaptation (1). Large shifts in the *B. pertussis* population resulted in antigenic divergence between circulating strains and vaccine strains (2–4). Further, in the 1980s, strains emerged with a novel allele for the pertussis toxin promoter *ptxP3,* replacing the resident *ptxP1* strains. The *ptxP3* strains produce more pertussis toxin than *ptxP1* strains and therefore may suppress host immunity more efficiently (5–7). More recently, strains deficient in the vaccine components pertactin (Prn) and filamentous hemagglutinin (FHA) were detected (8–15). Loss of one or both of these antigens may confer a selective advantage in vaccinated populations (16, 17). Here we present the completely closed and annotated genome sequences of 11 *B. pertussis* isolates which represent the pandemic *ptxP3* lineage and include six strains deficient in Prn and/or FHA.

Genomic DNA was isolated as described previously (18) and a 10-kb library was prepared. Sequencing was performed using a PacBio RS system with 6 single-molecule real-time (SMRT) cells per genome. The generated sequences were *de novo* assembled with HGAP (19) and trimmed and rotated by hand, resulting in a single circular contig for all genomes. *B. pertussis* genomes are highly similar and therefore RATT (20) was used to transfer annotations from *B. pertussis* Tohama I, CS, and 18323 (21–23). Afterward, sequenced genomes were manually checked for genes not present in the reference genomes.

The genomes comprised 4,100,705 to 4,111,557 bp and were predicted to have between 3,818 and 3,829 genes. Variation in the number of copies of the insertion sequence element IS*481*, which varied between 249 and 258 copies, was mainly responsible for the difference in gene numbers. The 11 strains were highly similar with respect to single nucleotide polymorphisms (SNPs) (*n* = 335) and small (up to 1,769 bp) insertions and deletions (*n* = 118). However, significant genome arrangements were observed, most likely mediated by insertion elements. Prn deficiency was caused by insertion of IS*481* in the *prn* gene (strains B3582, B3629, and B3640), a 25-bp deletion in the *prn* gene (strain B3621), or C-to-T mutation resulting in a stop codon in the *prn* gene (strain B3658). In one strain (B3582), FHA-deficiency was caused by insertion of a G in a homopolymeric tract of 10 Gs, leading to a premature translational termination. In the other FHA-deficient strain (B3585), no mutations in the *fhaB* gene*,* its promoter, or genes required for its surface expression were detected.

Comparisons of these strains and already published strains (18, 21, 22, 24) suggest that *B. pertussis* evolves not only by small mutations but also by major genome rearrangements which may affect gene regulation.

### Nucleotide sequence accession numbers.

The whole-genome shotgun projects have been deposited in DDBJ/ENA/GenBank under the accession numbers listed in Table 1. The versions described in this paper are the first versions.

**TABLE 1:** Characteristics of the 11 *B. pertussis* strains

Strain	Accession no.	Isolation yr	Country	*ptxP* type	*fim3* type	Prn^a^	FHA^a^
B1838	CP011440	1999	Netherlands	3	2	+	+
B1865	CP011441	1999	Netherlands	3	2	+	+
B3405	CP011442	2010	Netherlands	3	1	+	+
B3582	CP011443	2009	Sweden	3	2	−	−
B3585	CP011444	2009	Sweden	3	1	+	−
B3621	CP011401	2008	France	3	2	−	+
B3629	CP011400	2009	France	3	2	−	+
B3640	CP011445	2010	Netherlands	3	1	−	+
B3658	CP011446	2009	Norway	3	1	−	+
B3913	CP011447	2012	Netherlands	3	1	+	+
B3921	CP011448	2012	Netherlands	3	1	+	+

a +, strain produces Prn and/or FHA; −, strain does not produce Prn and/or FHA.
